# Adherence to Mass Drug Administration with Dihydroartemisinin–Piperaquine and *Plasmodium falciparum* Clearance in Southern Province, Zambia

**DOI:** 10.4269/ajtmh.19-0667

**Published:** 2020-07-02

**Authors:** Timothy P. Finn, Travis R. Porter, Hawela Moonga, Kafula Silumbe, Rachel F. Daniels, Sarah K. Volkman, Joshua O. Yukich, Joseph Keating, Adam Bennett, Richard W. Steketee, John M. Miller, Thomas P. Eisele

**Affiliations:** 1 Department of Tropical Medicine, Center for Applied Malaria Research and Evaluation, Tulane University School of Public Health and Tropical Medicine, New Orleans, Louisiana;; 2 National Malaria Elimination Centre, Zambia Ministry of Health, Chainama Hospital Grounds, Lusaka, Zambia;; 3 PATH Malaria Control and Elimination Partnership in Africa (MACEPA), Lusaka, Zambia;; 4 Harvard T.H. Chan School of Public Health, Boston, Massachusetts;; 5 The Broad Institute of MIT and Harvard, Cambridge, Massachusetts;; 6 Simmons University, Boston, Massachusetts;; 7 Malaria Elimination Initiative, Global Health Group, University of California San Francisco, San Francisco, California;; 8 PATH MACEPA, Seattle, Washington

## Abstract

Mass drug administration (MDA) with artemisinin combination therapy is a potentially useful tool for malaria elimination programs, but its success depends partly on drug effectiveness and treatment coverage in the targeted population. As part of a cluster-randomized controlled trial in Southern Province, Zambia evaluating the impact of MDA and household focal MDA (fMDA) with dihydroartemisinin–piperaquine (DHAp), sub-studies were conducted investigating population drug adherence rates and effectiveness of DHAp as administered in clearing *Plasmodium falciparum* infections following household mass administration. Adherence information was reported for 181,534 of 336,821 DHAp (53.9%) treatments administered during four rounds of MDA/fMDA, of which 153,197 (84.4%) reported completing the full course of DHAp. The proportion of participants fully adhering to the treatment regimen differed by MDA modality (MDA versus fMDA), RDT status, and whether the first dose was observed by those administering treatments. Among a subset of participants receiving DHAp and selected for longitudinal follow-up, 58 were positive for asexual-stage *P. falciparum* infection by microscopy at baseline. None of the 45 participants followed up at days 3 and/or 7 were slide positive for asexual-stage parasitemia. For those with longer term follow-up, one participant was positive 47 days after treatment, and two additional participants were positive after 69 days, although these two were determined to be new infections by genotyping. High completion of a 3-day course of DHAp and parasite clearance in the context of household MDA are promising as Zambia’s National Malaria Programme continues to weigh appropriate interventions for malaria elimination.

## INTRODUCTION

In the recent push to develop malaria elimination strategies, the Zambian government called for “high-impact malaria interventions” to sustain and improve on the success of malaria control strategies during the past few years.^1^ Among newly adopted strategies to reach elimination goals, Zambia’s National Malaria Elimination Centre has been implementing and evaluating the effectiveness and cost-effectiveness of mass drug administration (MDA) strategies with dihydroartemisinin–piperaquine (DHAp) across higher and lower transmission areas.^2,3^

The ability of MDA to achieve large-scale reductions in the prevalence of any infectious disease is a function of the population coverage achieved during a campaign and the drug’s effectiveness in clearing infections when implemented in a real-world setting. Previous studies comparing DHAp with other artemisinin-based combination therapies (ACTs) have shown DHAp to be highly efficacious at clearing asexual *Plasmodium falciparum* parasites.^4–11^ The long elimination half-life of piperaquine also offers up to 60 days of chemoprophylactic protection against new infections.^12,13^ Although evidence of delayed *P. falciparum* clearance and treatment failure among artemisinin-based therapies in general has been demonstrated in the Greater Mekong Subregion of Southeast Asia,^12,13^ limited reports of delayed clearance in the African continent do not currently demonstrate consistent artemisinin or ACT resistance.^14^

Using a highly efficacious drug such as DHAp, MDA effectiveness is dependent on the individual’s adherence to the medication regimen.^15,16^ When adherence to the full treatment regimen is incomplete, the antimalarial may not achieve or maintain therapeutic levels of the drug for a sufficient time to clear all parasites in an individual, placing selective pressure on surviving parasites.^17^ Low adherence rates across the treated population could fail to achieve maximum levels of effect and promote drug resistance, despite high treatment coverage in MDA.^18^ Studies across diverse settings in malaria and neglected tropical diseases have demonstrated wide variation in adherence during MDA activities.^15,16,19^ In a systematic review of adherence to malaria treatment, Bruxvoort et al.^15^ found adherence proportions varying as much as 11–100% across 30 descriptive studies. Therefore, it is reasonable to assume that a percentage of the population will not adhere fully to an MDA treatment program.^20^

As Zambia’s National Malaria Programme weighs the impact of MDA, additional insight into mediating issues, such as population-wide treatment adherence and the drug effectiveness in clearing parasites, would be useful for shaping expectations of MDA impact moving forward. To this end, this study presents results of analyses exploring issues of drug uptake and effectiveness. As part of the aforementioned mass treatment community-randomized controlled trial,^2^ we evaluated population acceptance and adherence to the DHAp regimen and parasite clearance during household MDA. Specifically, this study assesses population treatment adherence to DHAp under both community-wide MDA and fMDA modalities. We further examine reasons for adherence and nonadherence to treatment. Finally, to assess the effectiveness of DHAp as administered in this setting, we examine 3-day and 7-day parasite clearance among participants accepting treatment, as well as parasitemia at approximately 40 and 70 days following treatment.

## METHODS

A full description of the trial has been published elsewhere.^2^ In summary, the trial assessed the impact of community-wide MDA with DHAp or household-level focal MDA (fMDA), where DHAp was given to all eligible household members if anyone in the house had a positive malaria rapid diagnostic test (RDT; malaria *Pf* cassette test for histidine-rich protein 2 antigen [Standard Diagnostics Inc., Gyeonggi-do, Republic of Korea]). The trial engaged all households and residents of 60 health facility catchment areas covering a population of approximately 330,000 people in a region of heterogeneous transmission, with malaria prevalence in children ranging from < 1% to > 25% before the trial.^21^ The region is characterized by high coverage of vector control (long-lasting insecticide-treated mosquito nets and indoor residual spraying [IRS]) and good access via health facilities and community health workers (CHWs) to confirmed malaria case management with RDTs and artemether–lumefantrine (AL). The trial interventions included house-to-house campaigns of four rounds of RDT testing and DHAp treatment during both peak and nonpeak transmission seasons; rounds 1 through 4 occurred during November/December 2014 (nonpeak), February/March 2015 (peak), September/October 2015 (nonpeak), and February/March 2016 (peak), respectively.

### Treatment adherence.

During each campaign round, a pair of trained surveyors visited each household: a CHW who performed the testing and treatment for malaria and an enumerator who administered a standardized questionnaire to the head of the household or parental representative. Dihydroartemisinin–piperaquine formulations of 20 mg/160 mg and 40 mg/320 mg were provided per treatment modality to participants based on age according to the manufacturer’s recommendations. Pregnant women and children younger than 3 months were excluded from receiving DHAp. The full course of a single treatment was taken over three consecutive days and monitored using a modified directly observed therapy (DOT) strategy. On day 0 (baseline), the first dose of medication was administered and observed by the CHW and noted by the enumerator. The recipient was instructed to take the second dose at the same time the following day (day 1) but was unobserved. On day 2, a separate adherence officer visited the household to observe the third (final) dose, if possible, and to administer a follow-up questionnaire, irrespective of observing the final dose.

Initial questionnaires conducting during day 0 household visits recorded participant demographics, recent history of fever, household IRS exposure, recent travel, and bednet usage. At the end of each day, enumerators transferred name, age, gender, and a personal identifier for individuals who received DHAp to the adherence officer, blinded to testing status of individuals, for follow-up. The adherence questionnaire conducted on day 2 sought to ascertain the recipient’s testing and treatment recall, whether the dose was taken on the correct day, the number of tablets taken daily, reasons for not taking all doses, reasons for treatment refusal, and whether the recipient had heard MDA sensitization messages before the campaign visit. Visual inspection of the blister pack noted the number of remaining tablets, if any. These data were self-reported by the participants if aged 10 or older, or by the caregiver for those 9 years and younger. The person responding to the questions for the listed DHAp recipient was also recorded.

Assessment of treatment adherence used aggregated data from the four treatment rounds. An individual’s adherence status for a single course of drugs was classified into three categories: full adherence, partial adherence, and nonadherence. Full adherence was defined as taking all three doses and verifying that no tablets were remaining in the blister pack. Partial adherence was considered as taking one or two doses and/or having tablets remaining in the blister pack. Nonadherence was reporting having taken no doses. Although many people within the MDA treatment arm received up to four separate courses of DHAp over the study period, an individual’s adherence to treatments over multiple rounds was not assessed because it was not possible to link participants across treatment rounds.

Overall, adherence was compared across treatment arms by examining the proportion of full, partial, and nonadherence against key characteristics (e.g., high/low transmission, distribution round, gender, RDT test positivity, age, the number of RDT-positives per house, whether the first dose was directly observed by the CHW, and whether sensitization messages were heard). Reasons provided for nonadherence were assessed to determine the relative frequency of these responses. If responses coded as other were thematically similar to prelisted survey responses, these were recoded into the primary categories.

Multivariable logistic regression analysis was performed to assess whether demographics and/or household characteristics were associated with reporting of treatment adherence. A similar regression analysis was performed to assess factors associated with full adherence to DHAp treatment. Factors associated individually with full adherence in crude logistic models were added to a final multilevel regression model that also included random effects for catchment and treatment round.

### Parasite clearance.

For trial evaluation purposes, a subset of households in each of the control, MDA, and fMDA arms (260 households each) were enrolled in a concurrent longitudinal malaria incidence cohort beginning with the first treatment round in November 2014. These households were followed for 18 consecutive months—a period including all four treatment rounds. Cohort-enrolled households received the mass treatment intervention that coincided with their respective study arm at the same time as all other households within the area; however, visits to administer questionnaires, provide testing and treatment, and ascertain adherence to treatment were administered by local CHWs separately from campaign round households. In addition to RDT testing provided to campaign participants, all cohort participants had thick smear duplicate slides prepared for microscopy diagnosis and confirmation. Initial day 0 household questionnaires and day 2 adherence questionnaires were otherwise similar to those administered to households within the campaign areas but not enrolled in longitudinal follow-up.

As per protocol, participants in the longitudinal cohort with a positive RDT at enrollment (baseline visit; campaign round 1) and receiving an age-appropriate dose of DHAp were recruited to participate in a further sub-study to assess the effectiveness of DHAp, as administered via household MDA, in clearing parasites. To reduce the number of household visits, consenting participants were asked to present at the nearest health facility 3 and 7 days following the first dose of DHAp—corresponding to 1 and 4 days after completing the full course of DHAp, respectively.

At each follow-up facility visit, capillary blood was collected by finger stick and thick smear slides were prepared in duplicate. All slides were linked to respective cohort participants and sent to the National Malaria Elimination Centre for review. Two independent microscopists examined slides, specifically noting the presence and density of asexual- or sexual-stage *P. falciparum*. Other species were not consistently recorded. Results were fed into Microsoft Excel (Microsoft, Redmond, WA) spreadsheets. Discrepant readings were resolved by a third microscopist blinded to previous readings. Thin smear slides were not prepared to reduce the total number of slides prepared in the field.

In addition to the general exclusion criteria for receiving MDA mentioned earlier, individuals were excluded from parasite clearance assessment if questionnaires could not confirm receipt of DHAp. Following processing of blood slides, individuals with at least one thick blood smear positive for *P. falciparum* infection at baseline and receiving a course of DHAp were ultimately included in analyses. Short-term parasite clearance was estimated as the percentage of individuals present at follow-up who received a course of DHAp, had a microscopically confirmed *P. falciparum* infection at enrollment, and were negative for asexual *P. falciparum* (by microscopy) 3 and 7 days after the initial dose of treatment. Similarly, longer term parasite clearance was estimated as the percentage of individuals present at follow-up negative for asexual *P. falciparum* (by microscopy) approximately 1 and 2 months after treatment meeting the same criteria and also not receiving further treatment with antimalarials between baseline treatment and follow-up. For participants slide positive at long-term follow-up, molecular barcode analysis was performed on samples collected at baseline and follow-up (if available), as previously described,^22^ to determine whether *P. falciparum* infection at follow-up was likely due to infection with a new parasite or recrudescence of the initial infection. The study protocol was reviewed and ethical approval provided by the Research Ethics Committee of the University of Zambia, the Zambian Medicines Regulatory Authority, the Tulane University Institutional Review Board (IRB), and Western IRB.

## RESULTS

### Treatment adherence.

During the four campaign rounds, 336,821 DHAp treatments were provided to 383,768 eligible individuals, with a mean RDT positivity across the four campaign rounds of 5.1% (Table 1). Individuals who had not yet taken their final dose during the adherence visit because of dosage timing were excluded from this analysis (*n* = 1,309). Adherence teams completed follow-up visits for 181,534 (53.9%) of all 336,821 DHAp treatments. The proportion of treatments receiving follow-up visits ranged from 33.3% to 67.6% over the four rounds. There was no significant difference in adherence reporting between treatment arms overall (Pearson’s χ^2^, *P* = 0.99).

**Table 1:** Mass drug administration and fMDA household testing, treatment, and adherence reporting for each campaign treatment round

Category	Round 1		Round 2		Round 3		Round 4		Total
	fMDA	MDA	fMDA	MDA	fMDA	MDA	fMDA	MDA	
Households interviewed, *N*	17,704	18,237	14,610	14,584	18,004	17,528	16,050	15,257	131,975
Individuals listed, *N*	95,214	90,347	79,518	70,305	88,605	90,077	81,208	79,653	674,927
Tested for malaria, *n* (% listed)	79,774 (83.8%)	81,432 (90.1%)	67,566 (85.0%)	58,088 (82.6%)	79,389 (89.6%)	83,389 (92.6%)	74,493 (91.7%)	73,500 (92.3%)	597,631 (88.5%)
Rapid diagnostic test positivity, *n* (% tested)	7,866 (9.9%)	5,790 (7.1%)	3,797 (5.6%)	2,213 (3.8%)	4,509 (5.7%)	4,186 (5.0%)	1,475 (2.0%)	1,073 (1.5%)	30,898 (5.2%)
Eligible for treatment,* *N*	26,453	89,290	17,171	69,473	14,684	88,236	6,285	72,176	383,768
Treated with dihydroartemisinin–piperaquine, *n* (% eligible)	25,372 (95.9%)	78,591 (88.0%)	17,092 (99.5%)	56,620 (81.5%)	14,599 (99.4%)	72,006 (81.6%)	8,256 (131.4%)	64,285 (89.1%)	336,821 (87.8%)
Reporting adherence status, *n* (% treated)	13,847 (54.6%)	36,968 (47.0%)	7,461 (43.7%)	17,100 (30.2%)	8,507 (58.3%)	48,637 (67.5%)	5,375 (65.1%)	43,639 (67.9%)	181,534 (53.9%)
Full adherence, *n* (% reporting)	12,676 (91.5%)	29,800 (80.6%)	6,843 (91.7%)	14,467 (84.6%)	8,134 (95.6%)	39,197 (80.6%)	5,016 (93.3%)	37,054 (84.9%)	153,197 (84.4%)
Partial adherence, *n* (% reporting)	903 (6.5%)	5,974 (16.2%)	424 (5.7%)	2,095 (12.3%)	241 (2.8%)	7,373 (15.2%)	288 (5.4%)	5,141 (11.8%)	22,438 (12.4%)
Nonadherence, *n* (% reporting)	267 (1.9%)	1,194 (3.2%)	193 (2.6%)	539 (3.2%)	134 (1.6%)	2,067 (4.3%)	70 (1.3%)	1,444 (3.3%)	5,918 (3.3%)

fMDA = focal MDA; MDA = mass drug administration. Full adherence = all three doses completed; partial adherence = at least one dose, but not all completed; nonadherence = no doses taken.

* Household participants were eligible for treatment if residing in an area receiving MDA or residing within a household where at least one member tested positive for malaria in an area receiving fMDA.

Comparing characteristics of participants with and without adherence follow-up data, in adjusted analyses, individuals who reported fever in the last 2 weeks, were RDT positive, and had heard sensitization messages, and men were more likely to have been followed up and report adherence information (Table 2). Controlling for trial cluster and MDA round, the odds of reporting treatment adherence were higher among those who reported a fever within the previous 2 weeks, RDT positive, and indicated having heard MDA sensitization messages before the campaign. There were no differences in adherence follow-up and reporting by age, gender, or treatment arm.

**Table 2:** Crude and adjusted odds of reporting adherence information by campaign participant characteristic

Characteristic	*N*	Odds ratio (95% CI)	Adjusted odds ratio† (95% CI)
Age category (years):	336,794		
< 5		Ref.	Ref.
5–15		1.02* (1.01–1.05)	1.00 (0.98–1.02)
> 15		0.98* (0.96–0.99)	1.00 (0.98–1.01)
Gender (male)	327,918	1.03*** (1.02–1.05)	1.00 (0.99–1.02)
Fever in the previous 2 weeks	336,456	1.05* (1.01–1.10)	1.36*** (1.30–1.42)
RDT positive	331,905	1.01 (0.98–1.03)	1.17*** (1.14–1.20)
Treatment arm (MDA)	336,794	1.00 (0.98–1.02)	0.98 (0.70–1.37)
Heard sensitization messages	325,856	1.20*** (1.17–1.22)	1.11*** (1.09–1.13)

MDA = mass drug administration.

* *P* < 0.05, **P < 0.01, ***P < 0.001.

† Random effects for trial cluster and MDA rounds.

Among participants with adherence follow-up data, 153,197 of 181,534 (84.4%) across trial arms reported taking all three doses of DHAp (i.e., the full course), whereas 22,438 (12.4%) took only one or two doses (partially adherent) of the DHAp regimen (Table 1). Only 5,918 (3.3%) of participants reported taking no doses. Adherence status of individuals differed by trial arm with full adherence consistently greater in the fMDA trial arm than in the MDA arm across all four rounds (Pearson’s χ^2^, *P* = 0.001).

Adherence status also differed by DOT status during the first DHAp dose. When the first dose was directly observed by the CHW, full adherence was 83.6% in the MDA arm compared with 68.0% when it was not (Pearson’s χ^2^, *P* < 0.05). If the dose was taken the same day but after the CHW visit, full adherence was lower (78.0% in MDA and 86.5% in fMDA). If the treatment began any day after the initial visit, full adherence was 7.2% in MDA and 37.8% in fMDA.

A positive RDT result, residing in a cluster receiving fMDA, younger than 18 years, reporting hearing MDA sensitization messages, taking the first dose in front of a CHW, and self-responding to the adherence questions were significantly associated with an increase in the odds being fully adherent to treatment (Table 3). Conversely, having a fever within the previous 2 weeks was associated with decreased odds of full adherence (adjusted odds ratio: 0.85, 95% CI: 0.78–0.93, *P* < 0.001).

**Table 3:** Adjusted odds of full adherence

Regression covariate	Adjusted odds ratio† (95% CI)
Trial arm	
MDA	Ref.
FMDA	2.59** (1.31–5.15)
Person responding to adherence questions	
On behalf of someone who is absent	Ref.
On behalf of child younger than 10 years	1.50*** (1.43–1.59)
Self-response	2.00*** (1.92–2.09)
Rapid diagnostic test positive	1.30*** (1.22–1.39)
Age category (years)	
< 5	Ref.
5–15	1.00 (0.96–1.05)
> 15	0.80*** (0.75–0.85)
First treatment directly observed therapy by community health worker	1.35*** (1.28–1.42)
Heard MDA sensitization messages	1.17*** (1.12–1.22)
Gender (female)	1.03 (1.00–1.06)
Fever in the previous 2 weeks	0.85*** (0.78–0.93)
Total, *N*	170,453

fMDA = focal MDA; MDA = mass drug administration.

* *P* < 0.05, **P < 0.01, ***P < 0.001.

† Random effects for trial cluster and MDA rounds.

Among the 27,841 individuals classified as partially or completely nonadherent, 10,800 (39.3%) of these also reported reasons for their nonadherence. The primary reasons provided were as follows: 1) 8,909 (32.4%) individuals forgot, 2) 4,637 (16.8%) felt better, or 3) 2,558 (9.3%) lost medication. Only 1,369 (5.0%) reported having experienced side effects as the reason for stopping the treatment course early.

### Parasite clearance.

During the first treatment round, 1,567 participants from cohort households were reported as receiving a course of DHAp. Laboratory testing of 1,353 baseline thick blood smear slides identified 62 individuals with an asexual- or sexual-stage *P. falciparum* malaria infection at the time of treatment. Questionnaires indicated that 58 (93.5%) of these confirmed cases received DHAp. Baseline treatment was not reported or confirmed by adherence follow-up for the remaining four (6.5%) individuals who were excluded. Reasons for missing treatment data were not specified by data collectors, although two of these participants were from fMDA households where all household members were negative by RDT and, therefore, likely did not receive treatment as per trial protocol.

The number of days between baseline and follow-up household visits in later months varied among participants because of field-worker schedules for the cohort follow-up visits. First and second follow-up visits ranged from 21 to 63 (median: 44) days and 59 to 83 (median: 70) days after baseline, respectively. Three individuals tested positive by RDT during the first household visit and received further treatment with AL as per protocol for nonintervention months. Subsequent samples for these participants were excluded from analysis. Another participant reported having had a fever within the 2 weeks before the first follow-up visit but did not report taking an antimalarial during that period.

**Figure 1.**
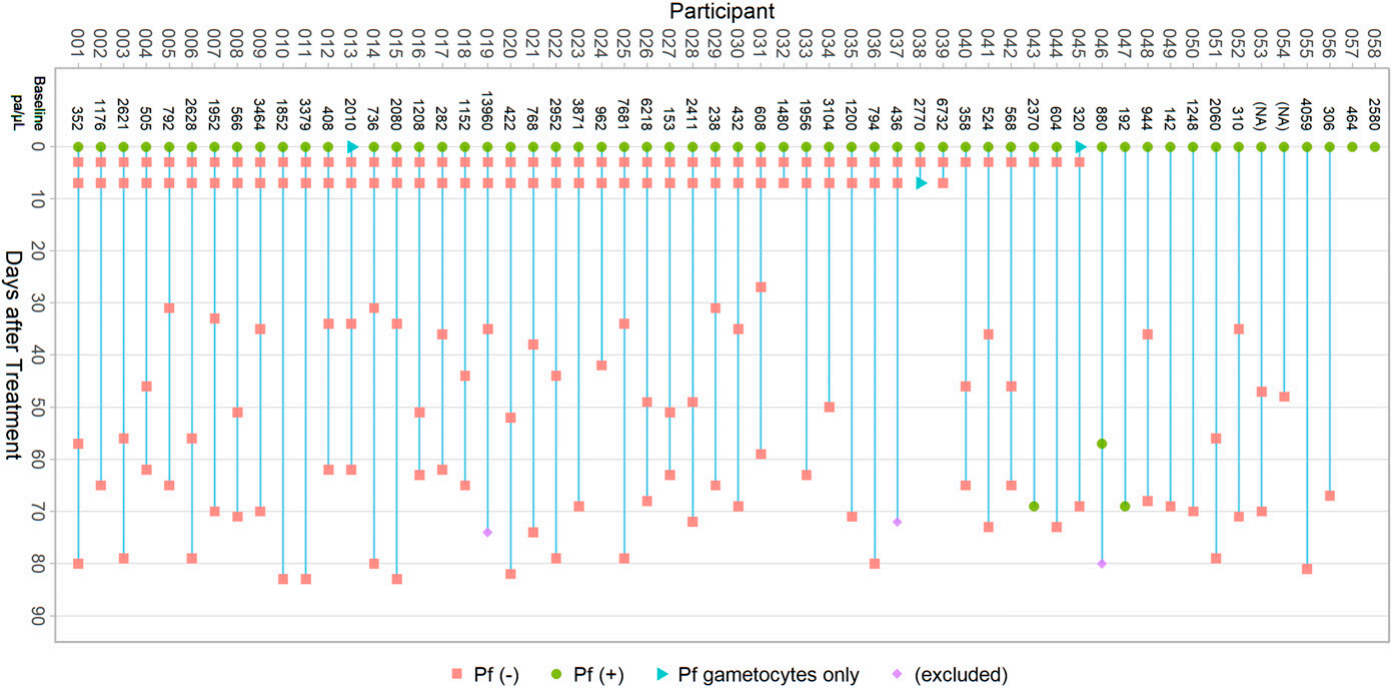
Clearance microscopy results. Baseline parasite density, timing of follow-up blood slide collection, and microscopy results for participants included in parasite clearance assessment. This figure appears in color at www.ajtmh.org.

Among those included in parasite clearance assessment at each time point, 45 of 58 (77.6%) and 39 of 58 (67.2%) presented to a local clinic 3 and 7 days after treatment for follow-up, respectively (Figure 1). Blood smears collected during follow-up household visits after initial treatment were available for 46 of 58 (79.3%) and 48 of 55 (87.3%) of (included) participants, respectively. Microscopy results were not available at any follow-up time point for two of 58 (3.4%) participants.

Table 4 provides the distribution of gender, age, and household treatment modality (fMDA or MDA) for confirmed infections at baseline and follow-up. No significant differences were seen in the distribution of characteristics between confirmed cohort infections and those presenting for clearance assessment or between participants at baseline and follow-up. Treatment adherence follow-up was recorded for 56 of 58 (96.6%) participants, all of whom reported taking the full course of DHAp.

**Table 4:** Characteristics of microscopy-confirmed *Plasmodium falciparum* infections and those eligible for clearance follow-up

Characteristic	Baseline		Follow-up			
	All cohort positive	Clearance eligible*	Day 3	Day 7	Month 1	Month 2
Baseline parasite density, mean pa/µL (95% CI)	1,795 (1,209–2,382)	1,861 (1,238–2,485)	2,023 (1,270–2,777)	2,213 (1,360–3,066)	1,855 (1,113–2,597)	1,518 (1,055–1,982)
Gender, % (95% CI)						
Female	54.8 (42.2–66.9)	55.2 (42.0–67.6)	57.8 (42.7–71.5)	61.5 (45.1–75.7)	56.5 (41.7–70.3)	62.5 (47.8–75.2)
Age category (years), % (95% CI)						
< 5	16.1 (8.8–27.7)	15.5 (8.2–27.5)	8.9 (3.3–21.9)	10.3 (3.8–25.0)	17.4 (8.8–31.5)	14.6 (7.0–28.0)
5–15	56.5 (43.7–68.4)	58.6 (45.4–70.7)	66.7 (51.4–79.1)	69.2 (52.7–82.0)	58.7 (43.7–72.2)	60.4 (45.7–73.4)
> 15	27.4 (17.6–40.0)	25.9 (16.1–38.9)	24.4 (13.9–39.4)	20.5 (10.4–36.5)	23.9 (13.5–38.7)	25.0 (14.6–39.5)
MDA treatment group, % (95% CI)						
Focal MDA	59.7 (46.9–71.3)	56.9 (43.7–69.2)	55.6 (40.6–69.6)	53.8 (37.8–69.1)	50.0 (35.6–64.4)	58.3 (43.7–71.6)
Mass drug administration	40.3 (28.7–53.1)	43.1 (30.8–56.3)	44.4 (30.4–59.4)	46.2 (30.9–62.2)	50.0 (35.6–64.4)	41.7 (28.4–56.3)
Total, *N*	62	58	45	39	46	48

Distribution of characteristics across time points was not significantly different by Pearson’s chi-square test.

* Participants eligible for analysis if confirmed positive by microscopy and reported as receiving treatment at baseline.

Microscopy results noted only gametocytes present at baseline for two of 58 (3.4%) participants. Parasite densities at baseline ranged from 142 to 13,960 (median: 1,056) parasites per μL. All available samples from days 3 and 7 were clear of asexual *P. falciparum* parasitemia (Table 5), although gametocytes were noted at day 7 for one participant. One participant was slide positive at the first household follow-up, 57 days after baseline. Two additional participants were slide positive at the second household follow-up, both 69 days after baseline DHAp treatment.

**Table 5:** Presence of *Plasmodium falciparum* parasites among participants throughout follow-up

Category	Baseline	Day 3	Day 7	Month 1*	Month 2†
Microscopy result, *n* (Col%)					
Negative	0 (0.0)	45 (100.0)	38 (97.4)	45 (97.8)	46 (95.8)
Positive	56 (96.6)	0 (0.0)	0 (0.0)	1 (2.2)	2 (4.2)
Gametocytes only	2 (3.4)	0 (0.0)	1 (2.6)	0 (0.0)	0 (0.0)
Total, *N*	58	45	39	46	48

* Follow-up visit ranged from 59 to 83 (median: 70) days after baseline.

† Follow-up visit ranged from 21 to 63 (median: 44) days after baseline.

Earlier parasite clearance could not be confirmed for the participant slide positive at day 57, as this individual did not appear for any short-term follow-up before this time. Polymerase chain reaction (PCR) confirmed infection at day 57, although lack of a baseline PCR sample precluded comparative genotyping to determine if this was indeed persistent infection or newly acquired. As mentioned earlier, this person was treated with AL at day 57 and subsequently clear of parasites by microscopy at day 80 (results excluded from Table 5). Among the two participants who were slide positive 69 days after treatment, the first was clear of parasites 3 days after baseline treatment. The second participant did not have short-term follow-up results but was slide negative at day 47. Both individuals resided in higher transmission clusters receiving fMDA and reported taking the full course of DHAp. Parasite genotyping analysis of baseline and day 69 samples from these individuals revealed that multiple positions of the 24 single nucleotide molecular bar code^12^ were distinct between pairs of samples, indicating that these were likely new parasite infections.

## DISCUSSION

The effectiveness of an infection control intervention is a function of whether the treatment is efficacious against the infection, the intervention reaches the target group, its users and providers adhere to intervention guidelines, and a high level of coverage is sustained over the necessary interval of time.^23,24^ As part of a trial of antimalarial MDA and fMDA compared with existing high-level coverage with standard of care interventions (the “high-intensity intervention package”),^25^ we examined the uptake and adherence to a 3-day drug regimen across multiple treatment rounds and explored factors associated with higher or lower levels of adherence. We also assessed the effectiveness of DHAp as administered in household MDA/fMDA for both short-term and durable clearance of *P. falciparum* infection. Overall, our evidence suggests acceptance and adherence to the DHAp regimen were high and maintained at these levels across all four mass treatment rounds, and that the drug was highly effective for short-term clearance of asexual parasite infection.

Adherence to a full course of DHAp observed across campaign rounds was high, with more than eight in 10 (84.4%) participants taking the full course of DHAp. Individuals in the fMDA areas, where treatment was provided to all members of a household when at least one person tested positive, were more than twice as likely to fully adhere as compared with those in the MDA arm. This difference was consistent across all four campaign rounds, irrespective of individual RDT status. These results could suggest that individuals in the fMDA trial arm recognized that if their household qualified for treatment they were at increased risk for malaria when they themselves were negative or sought to clear their infection when positive, whereas in MDA, where all individuals received treatment regardless of RDT positivity, full adherence was lower but constant across four rounds of MDA with declining RDT positivity in each successive round.

Of particular note, among campaign respondents in either of the MDA or fMDA trial arms with reported adherence data, 96.8% reported taking at least one dose of DHAp. The importance of taking all doses was emphasized by Hodel et al.^26^ in a modeling study of the optimal use of AL and DHAp in programmatic settings, which estimated parasite clearance rates at partial courses of DHAp. The predicted effects of taking all doses, missing the second dose, missing the third dose, and missing the second and third doses of DHAp had 91%, 70%, 71%, and 28% parasite clearance rates, respectively, for age-based dosing, but delaying a dose did not alter the treatment outcome.^26^ In areas where parasite transmission is low, such as the MDA trial arm in this study, the effect of not taking all doses may be attenuated in the context of a robust malaria control program with high vector control coverage for attaining elimination.^27^ Given that few participants eligible for clearance assessment reported incomplete adherence, this study did not have the power to produce clearance rate estimates for each category of adherence and, therefore, was not able support or contest the position of these modeling studies. Notwithstanding this limitation, efforts to improve full treatment adherence in MDA settings through improved community and individual sensitization of the parasite clearance and prophylactic benefits are necessary.

Integration of a parallel longitudinal cohort and early follow-up posttreatment allowed us to further assess clearance of *Pf* parasites after the initial MDA/fMDA round. DHAp contains two antimalarial drugs with differing elimination half-lives, and our assessment of parasite clearance considered both short- and long-term clearance, particularly important for longer lasting drugs.^28^ Among individuals with thick blood smear slides positive for *P. falciparum* infection and treated with an age-appropriate course of DHAp, all who were present for follow-up at day 3 and/or day 7 were free of asexual parasitemia. Two individuals (4.3%) visited 69 days following treatment were positive by microscopy. One individual was positive at day 57, although it was unclear whether this was due to late treatment failure or a new infection. These results are consistent with high clearance rates previously reported in sub-Saharan Africa.^7,8,10,29,30^

Genotyping indicated that the two late follow-up infections were likely newly acquired infections. The timing of these infections, between months 1 and 2 following treatment, is consistent with the waning prophylactic effect reported elsewhere.^12,13^ Distinguishing reinfection from recrudescence via genotyping has its limitations, however, particularly in higher transmission areas where infection with multiple *P. falciparum* genotypes are more likely.^28^ In cases of multiple infections before treatment, standard *msp*-1, *msp*-2, and *glurp* genotyping may miss genotypes present at baseline and misclassify the case as a reinfection at follow-up.^31,32^ The genome-wide SNP-based method used here aimed to provide better detection of mixed infections,^22^ with clear genotyping differences between alleles consistent with distinct infections. Genotyping findings were also supported by negative thick blood smears observed in the interim between baseline treatment and follow-up at 69 days. Both cases reported taking the full course of DHAp at baseline and occurred in higher transmission clusters where DHAp was only provided to households with a member testing positive by RDT (i.e., fMDA treatment clusters). fMDA may have left local infections untreated if undetected by RDTs during household visits, increasing the risk of reinfection for these individuals.

Although adherence data were collected for slightly more than half of the treatments provided, differences in demographic data between respondents were minor; consequently, it is believed that the adherence reporting and results herein provide a relatively unbiased estimate of adherence for the study area for the trial. Assessing individual adherence to medication that relies on self-reported histories may be prone to recall and social desirability bias. Evidence here suggested that individuals who were reporting on their own behavior were twice as likely to report full adherence compared with those who were not present and whose adherence was reported by another person. Furthermore, there are differences in how studies define full adherence.^15,20^ Here, we relied on self-reported histories (which is the most common assessment method), and to mitigate potential recall bias, we verified pill counts to ascertain physical proof of adherence. This too is prone to error because the absence of remaining medication is not confirmation that it was taken or taken correctly.^15^ However, there is limited evidence to suggest that the reporting is unreliable, and studies of adherence and accuracy of recall suggest that individuals do accurately recall their treatment during MDA scenarios.^33^

Challenges with conducting longitudinal follow-up across a large rural population limited availability of data and the ability to replicate the conditions of strict efficacy studies. The number of follow-up time points was fewer than those recommended in guidelines for efficacy studies.^34^ Inclusion of high transmission areas meant the possibility for a large number of potentially eligible participants. To balance the feasibility of collecting multiple samples across a rural area within a short period of time, recommended blood sample collection at 1, 2, and 14 days after treatment was dropped. As a result, the study was neither able to assess differences in clearance before day 3 nor identify late treatment failures before 1 month.

Recruitment of participants based on RDT results conducted during household visits affected availability of short-term follow-up data. Because microscopy results, needed to determine eligibility, were not available at the time of baseline household visits, individuals were invited to participate on RDT results alone and excluded those who were negative by RDT but later found to be positive by microscopy. Consequently, a relatively high number of slide-positive cohort individuals did not have follow-up results at days 3 and 7. This also resulted in differences in the proportion of participants in the clearance study available for assessment of short-term and long-term parasite clearance, as long-term follow-up used samples collected during monthly longitudinal cohort household visits. There was no evidence of statistically significant differences in demographic characteristics or study arm participation throughout follow-up, however. Additionally, the study only assessed clearance of *P. falciparum* parasitemia, as these infections are the most prevalent in this region. Although *Plasmodium malariae*, *Plasmodium ovale*, and *Plasmodium vivax* have been observed in this region, 97.5% of infections within this population during this time period were with *P. falciparum*.^35^

It is possible that long-term clearance follow-up participants sought additional treatment for malaria outside of the MDA campaign activities between baseline and follow-up visits. Data collectors recorded any treatments given during their visits and asked about recent history of fever and malaria treatment. The three participants receiving treatment for malaria roughly 1 month after baseline were excluded from analysis at later time points. Results reported here assume that no other malaria treatment was received during the period under analysis other than that already noted.

Despite challenges with conducting household treatment and adherence assessment over a large rural population, we were able to investigate components of mass treatment important to the overall impact of MDA conducting in an area of heterogeneous malaria transmission. The results reinforce the importance of community sensitization and mobilization before an MDA campaign, as well as directly observing the first dose. If testing is performed before drug administration, it appears that the household members will be significantly more likely to adhere to their treatment regimen if they or their family member test positive. Of note, the large sample size in our study likely led to finding statistically significant relationships with low effect estimates^36,37^; however, our estimates of determinants of adherence were consistent with other MDA studies and help characterize adherent from nonadherent individuals in this trial.^38^

In the context of a community-randomized controlled trial in Southern Province, Zambia, community-wide MDA and fMDA with DHAp both demonstrated high treatment adherence and high and durable clearance of malaria infections. Differences in adherence between MDA and fMDA trial arms suggest that the specific strategy for deploying an MDA intervention can influence the reception of treatment. In households where testing is performed and at least one person is positive (e.g., in our fMDA arm), this may be sufficient encouragement for other household members to complete a course of DHAp. In settings where no testing or selective testing is performed and no one is identified as positive (e.g., in some of the MDA households in our study), lower adherence may occur, especially where malaria risk is seen to be low. In such settings, additional efforts, such as community sensitization and full DOT, may be needed to ensure high treatment adherence. For microscopy-confirmed cases followed over time, DHAp provided a 100% short-term clearance of asexual *P. falciparum* parasite infections, with high PCR-adjusted clearance between 62 and 83 days after the DHAp treatment. Despite evidence of DHAp resistance outside of Africa, these results support the current utility of DHAp administered through MDA or fMDA rounds for effectively clearing asexual *P. falciparum* parasite infections in this setting.

## References

[1] Zambia Ministry of Health, 2012 National Malaria Control Programme Strategic Plan for FY 2011–2015: Consolidating Malaria Gains for Impact. Lusaka, Zambia: Ministry of Health, National Malaria Control Programme.

[2] Eisele TP 2015 Assessing the effectiveness of household-level focal mass drug administration and community-wide mass drug administration for reducing malaria parasite infection prevalence and incidence in Southern Province, Zambia: study protocol for a community randomized controlled trial. Trials 16: 347. 26268804 10.1186/s13063-015-0862-3 PMC4535296

[3] Yukich JO 2020 Cost-effectiveness of focal mass drug administration and mass drug administration with dihydroartemisinin-piperaquine for malaria prevention in Southern Province, Zambia: results of a community-randomized control trial. Am J Trop Med Hyg 103 (Suppl 2): 46–53. 32618249 10.4269/ajtmh.19-0661 PMC7416981

[4] Mayxay M 2006 An open, randomized comparison of artesunate plus mefloquine vs. dihydroartemisinin-piperaquine for the treatment of uncomplicated *Plasmodium falciparum* malaria in the Lao People’s Democratic Republic (Laos). Trop Med Int Health 11: 1157–1165. 16903879 10.1111/j.1365-3156.2006.01671.x

[5] Ashley EA 2005 A randomized, controlled study of a simple, once-daily regimen of dihydroartemisinin-piperaquine for the treatment of uncomplicated, multidrug-resistant falciparum malaria. Clin Infect Dis 41: 425–432. 16028147 10.1086/432011

[6] Trung TN Tan B Van Phuc D Song J, 2009 A randomized, controlled trial of artemisinin-piperaquine vs dihydroartemisinin-piperaquine phosphate in treatment of falciparum malaria. Chin J Integr Med 15: 189–192. 19568711 10.1007/s11655-009-0189-6

[7] Ogutu BR 2014 Efficacy and safety of artemether-lumefantrine and dihydroartemisinin-piperaquine in the treatment of uncomplicated *Plasmodium falciparum* malaria in Kenyan children aged less than five years: results of an open-label, randomized, single-centre study. Malar J 13: 33. 24472156 10.1186/1475-2875-13-33 PMC3916309

[8] Sylla K 2013 Monitoring the efficacy and safety of three artemisinin based-combinations therapies in Senegal: results from two years surveillance. BMC Infect Dis 13: 598. 24354627 10.1186/1471-2334-13-598 PMC3878220

[9] Song J 2011 Randomized trials of artemisinin-piperaquine, dihydroartemisinin-piperaquine phosphate and artemether-lumefantrine for the treatment of multi-drug resistant falciparum malaria in Cambodia-Thailand border area. Malar J 10: 231. 21827706 10.1186/1475-2875-10-231 PMC3169515

[10] Zwang J 2009 Safety and efficacy of dihydroartemisinin-piperaquine in falciparum malaria: a prospective multi-centre individual patient data analysis. PLoS One 4: e6358. 19649267 10.1371/journal.pone.0006358 PMC2716525

[11] Four Artemisinin-Based Combinations (4ABC) Study Group, 2011 A head-to-head comparison of four artemisinin-based combinations for treating uncomplicated malaria in African children: a randomized trial. PLoS Med 8: e1001119. 22087077 10.1371/journal.pmed.1001119 PMC3210754

[12] Sawa P 2013 Malaria transmission after artemether-lumefantrine and dihydroartemisinin-piperaquine: a randomized trial. J Infect Dis 207: 1637–1645. 23468056 10.1093/infdis/jit077

[13] Zani B Gathu M Donegan S Olliaro PL Sinclair D, 2014 Dihydroartemisinin-piperaquine for treating uncomplicated *Plasmodium falciparum* malaria. Cochrane Database Syst Rev 1: 1–160. 10.1002/14651858.CD010927 PMC4470355 24443033

[14] World Health Organization, 2018 Status Report on Artemisinin Resistance and ACT Efficacy (August 2018). Available at: http://www.who.int/malaria/publications/atoz/artemisinin-resistance-august2018/en/. Accessed November 29, 2018.

[15] Bruxvoort K Goodman C Kachur SP Schellenberg D, 2014 How patients take malaria treatment: a systematic review of the literature on adherence to antimalarial drugs. PLoS One 9: e84555. 24465418 10.1371/journal.pone.0084555 PMC3896377

[16] Krentel A Fischer PU Weil GJ, 2013 A review of factors that influence individual compliance with mass drug administration for elimination of lymphatic filariasis. PLoS Negl Trop Dis 7: e2447. 24278486 10.1371/journal.pntd.0002447 PMC3836848

[17] White NJ Pongtavornpinyo W Maude RJ Saralamba S Aguas R Stepniewska K Lee SJ Dondorp AM White LJ Day NP, 2009 Hyperparasitaemia and low dosing are an important source of anti-malarial drug resistance. Malar J 8: 253. 19906307 10.1186/1475-2875-8-253 PMC2784792

[18] Gosling RD Okell L Mosha J Chandramohan D, 2011 The role of antimalarial treatment in the elimination of malaria. Clin Microbiol Infect 17: 1617–1623. 21951597 10.1111/j.1469-0691.2011.03660.x

[19] Shuford KV Turner HC Anderson RM, 2016 Compliance with anthelmintic treatment in the neglected tropical diseases control programmes: a systematic review. Parasit Vectors 9: 29. 26813098 10.1186/s13071-016-1311-1 PMC4729159

[20] Yeung S White NJ, 2005 How do patients use antimalarial drugs? A review of the evidence. Trop Med Int Health 10: 121–138. 15679555 10.1111/j.1365-3156.2004.01364.x

[21] Eisele TP 2016 Short-term impact of mass drug administration with dihydroartemisinin plus piperaquine on malaria in Southern Province Zambia: a cluster-randomized controlled trial. J Infect Dis 214: 1831–1839. 27923947 10.1093/infdis/jiw416 PMC5142084

[22] Daniels R 2008 A general SNP-based molecular barcode for *Plasmodium falciparum* identification and tracking. Malar J 7: 223. 18959790 10.1186/1475-2875-7-223 PMC2584654

[23] Tanner M Lengeler C Lorenz N, 1993 From the efficacy of disease control tools to community effectiveness. Trans R Soc Trop Med Hyg 87: 518–523. 8266400 10.1016/0035-9203(93)90070-7

[24] Littrell M Miller JM Ndhlovu M Hamainza B Hawela M Kamuliwo M Hamer DH Steketee RW, 2013 Documenting malaria case management coverage in Zambia: a systems effectiveness approach. Malar J 12: 371. 24160186 10.1186/1475-2875-12-371 PMC3842626

[25] Eisele TP 2020 Impact of four rounds of mass drug administration with dihydroartemisinin-piperaquine implemented in Southern Province, Zambia. Am J Trop Med Hyg 103 (Suppl 2): 7–18. 10.4269/ajtmh.19-0659 PMC7416977 32618247

[26] Hodel EM Kay K Hayes DJ Terlouw DJ Hastings IM, 2014 Optimizing the programmatic deployment of the anti-malarials artemether-lumefantrine and dihydroartemisinin-piperaquine using pharmacological modelling. Malar J 13: 138. 24708571 10.1186/1475-2875-13-138 PMC4036747

[27] Nikolov M Bever CA Upfill-Brown A Hamainza B Miller JM Eckhoff PA Wenger EA Gerardin J, 2016 Malaria elimination campaigns in the Lake Kariba region of Zambia: a spatial dynamical model. PLoS Comput Biol 12: e1005192. 27880764 10.1371/journal.pcbi.1005192 PMC5120780

[28] Stepniewska K White NJ, 2006 Some considerations in the design and interpretation of antimalarial drug trials in uncomplicated falciparum malaria. Malar J 5: 127. 17187673 10.1186/1475-2875-5-127 PMC1769507

[29] World Health Organization, 2016 Status Report on Artemisinin and ACT Resistance (October 2016). Available at: http://www.who.int/malaria/publications/atoz/update-artemisinin-resistance-october2016/en/. Accessed November 1, 2017.

[30] Muhindo MK 2014 Early parasite clearance following artemisinin-based combination therapy among Ugandan children with uncomplicated *Plasmodium falciparum* malaria. Malar J 13: 32. 24468007 10.1186/1475-2875-13-32 PMC3909240

[31] Juliano JJ Ariey F Sem R Tangpukdee N Krudsood S Olson C Looareesuwan S Rogers WO Wongsrichanalai C Meshnick SR, 2009 Misclassification of drug failures in *Plasmodium falciparum* clinical trials in southeast Asia. J Infect Dis 200: 624–628. 19591576 10.1086/600892 PMC2761972

[32] Juliano JJ Gadalla N Sutherland CJ Meshnick SR, 2010 The perils of PCR: can we accurately ‘correct’ antimalarial trials? Trends Parasitol 26: 119–124. 20083436 10.1016/j.pt.2009.12.007 PMC2844636

[33] Budge PJ Sognikin E Akosa A Mathieu EM Deming M, 2010 Accuracy of coverage survey recall following an integrated mass drug administration for lymphatic filariasis, schistosomiasis, and soil-transmitted helminthiasis. PLoS Negl Trop Dis 10: e0004358. 10.1371/journal.pntd.0004358 PMC4713198 26766287

[34] World Health Organization, 2003 Assessment and Monitoring of Antimalarial Drug Efficacy for the Treatment of Uncomplicated Falciparum Malaria. Available at: https://www.who.int/malaria/publications/atoz/whohtmrbm200350/en/. Accessed November 28, 2018.

[35] Chishimba S 2020 Prevalence of *Plasmodium falciparum* and non-*falciparum* infections by photo-induced electron transfer-PCR in a longitudinal cohort of individuals enrolled in a mass drug administration trial in Southern Province, Zambia. Am J Trop Med Hyg 103 (Suppl 2): 82–89. 32618252 10.4269/ajtmh.19-0668 PMC7416980

[36] Kalinowski P Fidler F, 2010 Interpreting significance: the differences between statistical significance, effect size, and practical importance. Newborn Infant Nurs Rev 10: 50–54.

[37] Kühberger A Fritz A Lermer E Scherndl T, 2015 The significance fallacy in inferential statistics. BMC Res Notes 8: 84. 25888971 10.1186/s13104-015-1020-4 PMC4377068

[38] Yakasai AM Hamza M Dalhat MM Bello M Gadanya MA Yaqub ZM Ibrahim DA Hassan-Hanga F, 2015 Adherence to artemisinin-based combination therapy for the treatment of uncomplicated malaria: a systematic review and meta-analysis. J Trop Med 2015: 189232. 26161095 10.1155/2015/189232 PMC4464595

